# Supplementary material for: The Optimization of Extraction Process, Antioxidant, Whitening and Antibacterial Effects of Fengdan Peony Flavonoids
Source: Molecules. 2022 Jan 14;27(2):506. doi: 10.3390/molecules27020506 (PMC8780704; doi:10.3390/molecules27020506)
Supplement: Supplementary file 1 [file molecules-27-00506-s001.zip › molecules-1520053-supplementary.pdf]

Supplementary Material

The optimization of extraction process, antioxidant, whitening and antibacterial effects of Fengdan peony flavonoids

Jie Lu[+], Zhiqiang Huang[+], Yusheng Liu, Huimin Wang, Min Qiu, Yinghui Qu, Wenpeng Yuan*

Heze Branch, Qilu University of Technology (Shandong Academy of Sciences), Biological Engineering Technology Innovation Center of Shandong Province, Heze, 274000, China;

lujiesunshine@163.com (J.L.); hzqlucky@163.com (Z.H.); liuyusheng666@126.com (Y.L.); whm_hy@sina.com (H.W.); qmqh2018@163.com (M.Q.); QYH9020@163.com (Y.Q.);

*Correspondence: sdashz@163.com;

[+] These authors contributed equally to this work.

Table S1. The design and results of RSM experiment.

NO.	A	B	C	Total flavonoidss yield (%)
1	-1	0	1	1.150
2	0	0	0	1.191
3	0	1	-1	1.110
4	1	1	0	1.126
5	0	0	0	1.184
6	-1	0	-1	1.114
7	1	-1	0	1.153
8	0	-1	-1	1.160
9	0	-1	1	1.136
10	-1	1	0	1.046
11	0	0	0	1.197
12	0	1	1	1.112
13	1	0	-1	1.167
14	-1	-1	0	1.145
15	1	0	1	1.119
16	0	0	0	1.178
17	0	0	0	1.197

Table S2. The MIC of flavonoids to 4 kinds of Gram-positive bacteria

**Antibacterial concentration（mg/ml）**	***S.aureus***	***B.anthracis***	***B.subtilis***	***C.perfringens***
0.0037	+	+	+	+
0.0073	+	+	+	+
0.0146	+	+	+	+
0.0293	-	+	+	+
0.0586	-	+	+	+
0.1172	-	-	+	+
0.2344	-	-	-	+
0.4688	-	-	-	+
0.9375	-	-	-	+
1.8750	-	-	-	+
3.7500	-	-	-	+
7.500	-	-	-	-
15.000	-	-	-	-
Positive control	-	-	-	-
Negative control	+	+	+	+

Note: “-” means aseptic growth, “+” means bacterial growth.

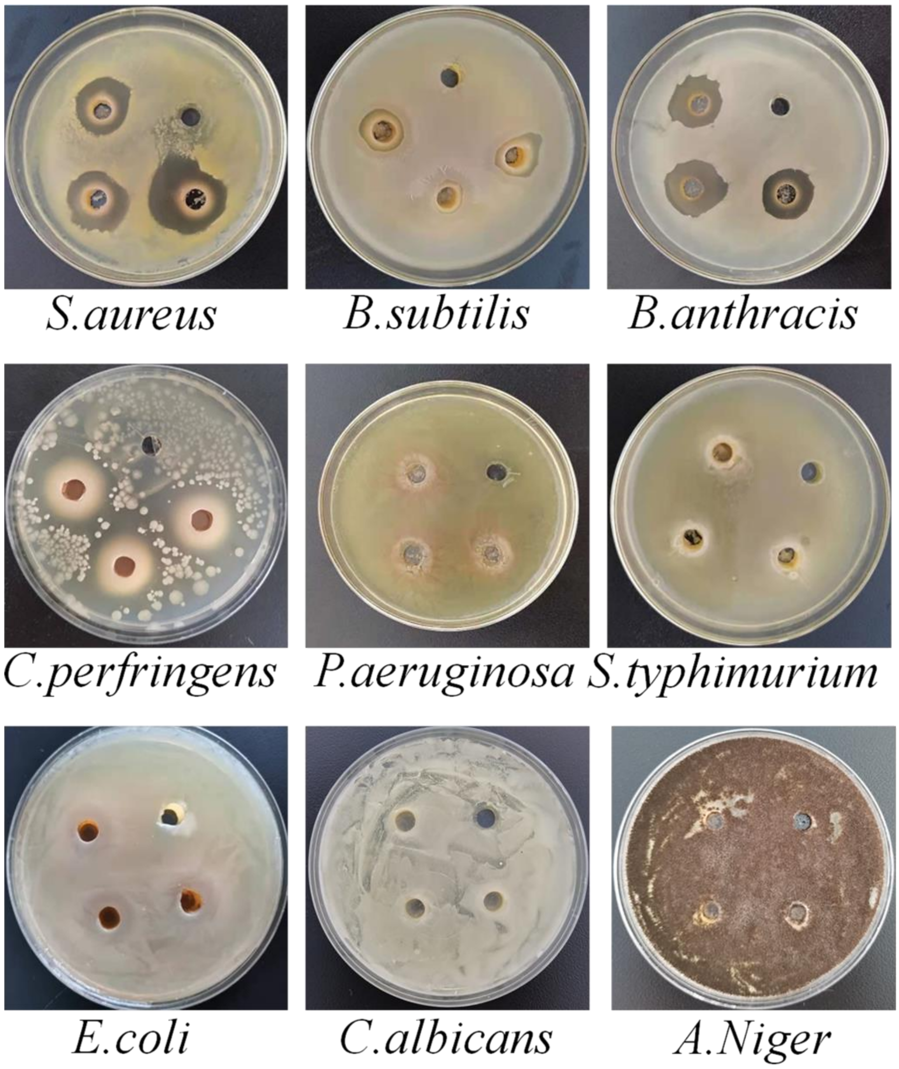

Figure S1. The results of inhibition zone experiment for 30 mg/mL peony. total flavonoids to 9 kinds of bacteria.
